# Exploring the Association Between Medically Assisted Reproduction and Autism Spectrum Disorder: Clinical Correlations from a Retrospective Cohort

**DOI:** 10.3390/pediatric17060118

**Published:** 2025-11-04

**Authors:** Federica Gigliotti, Maria Eugenia Martelli, Silvia Foglietta, Alessia Balestrini, Carla Sogos

**Affiliations:** Department of Human Neuroscience, Sapienza University of Rome, 00185 Rome, Italy; federica.gig@uniroma1.it (F.G.); mariaeugenia.martelli@uniroma1.it (M.E.M.); silvia.foglietta@uniroma1.it (S.F.);

**Keywords:** assisted reproductive technology (ART), autism spectrum disorder (ASD), neurodevelopmental disorders (NDDs), perinatal outcomes, risk factors

## Abstract

**Background/Objectives**: Autism Spectrum Disorder (ASD) is a neurodevelopmental condition characterized by impairments in social interaction and communication, as well as by repetitive behaviors, with a rising global prevalence. Concurrently, the use of Assisted Reproductive Technologies (ART) has increased among couples experiencing infertility. This study aimed to compare the frequency of ART-conceived children between those diagnosed with ASD and those with other neurodevelopmental disorders (nASD), and to examine differences in prenatal, perinatal and medical histories of ART- and spontaneously (non-ART)-conceived children within an ASD group. **Methods**: We retrospectively analyzed data from 507 children with a neurodevelopmental disorders (NDDs) diagnosis, classified into ASD (*n* = 234) and nASD (*n* = 273) groups. Subsequent analyses focused on the ASD group, further divided into an ART and non-ART group according to the conception mode. **Results**: ART-conceived children were more frequent in the ASD group than in the nASD group. Moreover, within ASD, ART was significantly associated with potential risk factors such as twin pregnancy, cesarean delivery, low birth weight and parental age. Logistic Binary Regression confirmed these results, suggesting that ART co-occurs with a cluster of perinatal and familial risk factors. **Conclusions**: Our results indicate that ART is not an independent causal exposure; however, given the retrospective design and the absence of a general population control group, causal inference cannot be drawn. The observed association with ASD appears to be mediated by perinatal and parental variables. These findings underscore the importance of improving obstetric management and care, and ensuring early developmental monitoring for ART-conceived children.

## 1. Introduction

Neurodevelopmental disorders (NDDs), including autism spectrum disorder (ASD), language disorder (LD), and mixed developmental disorder (MDD), are heterogeneous conditions that typically emerge in early childhood and persist throughout the lifespan. These disorders affect multiple domains of functioning, such as communication, social interaction, cognition, and adaptive behavior, and impose a substantial burden on individuals, families, and healthcare systems [1,2]. Epidemiological evidence indicates that ASD alone affects approximately 1–2% of the global population, with higher prevalence estimates in clinical referral cohorts [3]. Given the long-term service needs and economic impact associated with NDDs, clarifying risk factors is a priority for both research and clinical practice [4].

The etiology of NDDs is complex and multifactorial, reflecting interactions between genetic predispositions, epigenetic mechanisms, and environmental exposures during sensitive developmental windows [5]. Among environmental factors, assisted reproductive technologies (ART) have attracted increasing interest. ART refers to medical procedures such as in vitro fertilization (IVF) and intracytoplasmic sperm injection (ICSI), which have transformed reproductive medicine by offering new possibilities for couples experiencing infertility [6]. In recent decades, ART use has risen markedly worldwide, with over 8 million children conceived using these procedures [7]. In Europe, ART accounts for 3–5% of all births, with comparable figures reported in Italy [8].

While ART has provided reproductive opportunities, concerns have been raised about potential long-term consequences for offspring. A substantial body of evidence shows that ART is associated with increased risks of adverse obstetric and perinatal outcomes, including multiple pregnancies, preterm birth, low birth weight, and cesarean delivery [9,10]. These complications are observed not only in multiple but also in singleton ART pregnancies, suggesting that risks may be related to ART procedures themselves or to underlying parental factors [11]. Since perinatal adversities such as preterm birth and intrauterine growth restriction are recognized predictors of atypical neurodevelopment [12], ART has become a focus of research into its possible association with NDDs, especially ASD.

Several systematic reviews and meta-analyses have investigated this association, although the findings remain inconsistent. Epidemiological studies suggest that ART may be associated with an increased risk of ASD, with relative risks ranging from 1.2 to 1.4 compared to natural conception [13,14]. Liu et al. reported a 35% increased risk of ASD in ART-conceived children (RR = 1.35, 95% CI: 1.09–1.68) [13], while Andreadou et al. found a modest association (RR = 1.11, 95% CI: 1.03–1.19) that disappeared when analyses were restricted to singletons [15]. By contrast, large-scale register-based studies, such as those conducted in Nordic countries, have not consistently confirmed this association after adjustment for parental and perinatal variables [11].

A recent systematic review confirmed that ART pregnancies are associated with markedly higher rates of multiple births (RR = 14.57), preterm birth (RR = 3.83), and low birth weight (RR = 5.66) compared to natural conception [14]. These outcomes represent important mediators in the relationship between ART and neurodevelopmental outcomes. Indeed, studies show that the apparent increased risk of ASD in ART-conceived children is significantly attenuated once obstetric complications and multiple pregnancies are accounted for [16]. Winter et al. demonstrated that multiple pregnancy, preterm birth, and cesarean delivery collectively explained nearly 78% of the ART–ASD association, underscoring the central role of perinatal mediators [17].

Parental age represents another critical factor. Couples who undergo ART are typically older at conception than those conceiving naturally, reflecting broader demographic trends. Advanced parental age, particularly paternal age, has been independently associated with increased ASD risk. Large-scale cohort studies report hazard ratios ranging from 1.39 to 3.45 for advanced paternal age [18,19]. Idring et al. showed that paternal age above 50 years was associated with more than a threefold increased risk of ASD [18], while D’Onofrio et al. confirmed similar associations, extending the findings to a broader range of psychiatric and developmental outcomes [19]. Thus, parental age may act as both a confounder and a mediator in the ART–ASD relationship.

Taken together, the literature offers a complex picture. Registry-based studies provide large sample sizes but often lack diagnostic precision and detailed family history, whereas clinical cohort studies yield richer clinical data but are comparatively scarce and smaller in scale. Moreover, there is limited evidence from the Italian context, despite ART being increasingly common in Southern Europe.

The present study was designed to address these gaps by analyzing a large, clinically characterized cohort of children referred to a tertiary outpatient service. The aims were (1) to compare the prevalence of ART conception between children diagnosed with ASD and those with other NDDs (nASD); and (2) within the ASD subgroup, to investigate associations between ART conception and prenatal, perinatal, and familial factors.

From a clinical perspective, clarifying this relationship has important implications. If ART is not an independent risk factor but a marker of perinatal risk, preventive efforts should focus on optimizing obstetric management and developmental monitoring in ART-conceived children. Alternatively, if ART is directly associated with neurodevelopment, fertility counseling should incorporate potential long-term risks. In both scenarios, disentangling the interplay between ART, parental age, and perinatal outcomes provides valuable guidance for clinicians, policymakers, and families, supporting early identification and intervention strategies for children at risk.

## 2. Materials and Methods

### 2.1. Participants

The study analyzed pre-, perinatal and medical history data of 507 patients referred to the Outpatient Service for Neurodevelopmental Disorders, Department of Human Neuroscience, Sapienza University of Rome, up to February 2025. Inclusion criteria were: (a) a diagnosis of Neurodevelopmental Disorder (NDD) according to the International Classification of Diseases, 10th Revision (ICD-10), currently adopted in the Italian national healthcare system; and (b) availability of information on conception, including the use of assisted reproductive technology (ART).

The sample included 408 males and 99 females (sex ratio ≈ 4:1). Diagnoses were autism spectrum disorder (ASD, *n* = 234; 46.2%), mixed developmental disorder (MDD, *n* = 179; 35.3%), and language disorder (LD, *n* = 94; 18.5%). Patients without an ASD diagnosis were classified as non-ASD (nASD, *n* = 273).

Overall, 47 children were conceived through ART (32 males, 15 females): 31 with ASD, 7 with MDD, and 9 with LD. The distribution of ART and non-ART conceptions by diagnostic group is reported in Table 1.

### 2.2. Procedure

This retrospective study was designed to explore the association between ART and ASD within a clinically referred cohort of children with NDDs. To this end, participants were divided into two diagnostic groups: ASD and nASD.

Clinical information was systematically collected from medical records across four domains: (a) general data (sex, age at evaluation, and ICD-10 NDD diagnosis); (b) pregnancy history (mode of conception [natural vs. ART], singleton/multiple pregnancy, complications); (c) perinatal history (delivery mode [vaginal vs. cesarean], gestational age at birth, neonatal status, birth weight); and (d) family history (maternal/paternal age at delivery, history of miscarriage and recurrent miscarriage [≥2], maternal medication or medical conditions during pregnancy, family history of neuropsychiatric disorders on maternal and/or paternal side, and siblings with neuropsychiatric disorders).

### 2.3. Data Analysis

All statistical analyses were performed using jamovi software version 2.5.5 (The jamovi project, Sydney, Australia, 2024). To facilitate the analyses, all variables except continuous measures (age and weight) were coded dichotomously (0 = absent, 1 = present). Sex was coded as male = 1 and female = 0.

Categorical variables included: sex, ART conception, twin pregnancy, uncomplicated pregnancy, vaginal delivery (present/absent, with “absent” indicating cesarean section delivery), preterm birth (<37 weeks), neonatal well-being, low birth weight (<2500 g), history of miscarriage, recurrent miscarriages (≥2), maternal medical conditions, maternal medication during pregnancy, family history of neuropsychiatric disorders (any, maternal side, paternal side, siblings).

Continuous variables included: gestational age at birth (weeks), age at assessment (months), birth weight (grams), maternal age at delivery (years), and paternal age at delivery (years).

Normality was assessed using the Shapiro–Wilk test and visual inspection of Q-Q plots and histograms. Maternal and paternal age were normally distributed, while gestational age at birth, birth weight and age at assessment were not. For descriptive purposes, continuous variables were presented as mean ± standard deviation (SD), regardless of distribution, and dichotomous variables as count or percentage.

Group comparisons were conducted using χ^2^ tests for categorical variables, Student’s *t*-tests for normally distributed continuous variables, and Mann–Whitney U tests for non-normal variables. Pearson’s correlation was applied to compute the correlation matrix across all variables. For pairs of continuous variables the correlation coefficient corresponded to standard Pearson’s *r*; for associations between continuous and dichotomous (0/1) variables it was equivalent to the point-biserial correlation coefficient; and for associations between dichotomous variables, the *r* coefficient corresponded to the φ (phi). For clarity the coefficients were reported as *r*. Binary logistic regression models and relative risk (RR) estimates were used to examine the potential relationships between ART and obstetric, perinatal, or familial factors, controlling for maternal and paternal age.

Statistical significance was set at *p* < 0.05. Bonferroni correction was applied for multiple comparisons to reduce the risk of type I error.

## 3. Results

### 3.1. Distribution of Assisted Reproductive Technology

We compared the prevalence of ART conception between the ASD and nASD groups. A chi-square test showed a significant difference (χ^2^ = 8.17, *p* = 0.004): 13.2% of children with ASD versus 5.9% of non-ASD children were conceived via ART. The relative risk indicated that ART-conceived children had a 64% higher risk of receiving an ASD diagnosis compared with naturally conceived children and the corresponding odds ratio was 2.45 (RR = 1.64, 95% CI 1.09–2.46; OR = 2.4, 95% CI 1.31–4.61 Table 2).

### 3.2. Medical History of the ASD Group

The ASD group included 234 children (185 males, 49 females) of whom 31 (13.2%) were conceived via ART.

Perinatal characteristics showed a mean gestational age of 38.6 weeks (SD = 2.3) and a mean birth weight of 3189 g (SD = 592). Low birth weight (<2500 g) was recorded in 26 children (11.1%), and 33 (14.1%) were born preterm. Cesarean delivery occurred in 131 cases (56.0%), and twin pregnancy in 19 (8.1%). Neonatal well-being was reported in 192 children (82.1%).

Regarding family history, the mean paternal and maternal ages at delivery were 36.7 years (SD = 6.43) and 33.6 years (SD = 6.32), respectively. A history of miscarriage was reported in 28 mothers (12.0%), and 47 mothers (20.1%) reported medication use during pregnancy. Maternal medical conditions were present in 66 cases (28.2%).

A family history of neuropsychiatric disorders was reported in 106 children (45.3%), with similar frequencies on the maternal (65 cases) and paternal sides (61 cases). Siblings with neuropsychiatric disorders were reported in 18 cases (7.7%). Detailed distributions by ART and non-ART conception are presented in Table 3.

### 3.3. Association Between ART and Risk Factors Within ASD Group: Correlation Analysis and Regression Analysis

Within the ASD group, ART conception was positively correlated with twin pregnancy (*r* = 0.30, *p* < 0.001), low birth weight (*r* = 0.18, *p* = 0.005), and recurrent miscarriage (*r* = 0.15, *p* = 0.019), and negatively correlated with vaginal delivery (*r* = −0.22, *p* < 0.001). ART was also strongly associated with higher maternal (*r* = 0.47, *p* < 0.001) and paternal age (*r* = 0.33, *p* < 0.001).

Twin pregnancy, in turn, was linked to preterm birth (*r* = 0.51, *p* < 0.001), low birth weight (*r* = 0.49, *p* < 0.001), higher maternal age (*r* = 0.19, *p* = 0.004), and lower rates of vaginal delivery (*r* = −0.26, *p* < 0.001) and uncomplicated pregnancy (*r* = −0.13, *p* = 0.04).

Uncomplicated pregnancy was inversely related to preterm birth, low birth weight, maternal medical conditions, and maternal medication use during pregnancy (all *r* ≈ −0.18 to −0.22, *p* ≤ 0.006), and positively associated with neonatal well-being (*r* = 0.21, *p* = 0.001). Neonatal well-being was negatively correlated with low birth weight (*r* = −0.44, *p* < 0.001).

Correlational analyses (Pearson’s *r*, equivalent to phi or point-biserial for binary variables) were used to explore associations between risk factors within the ASD group. Given their exploratory nature, no corrections for multiple testing were applied; results should therefore be considered hypothesis-generating and interpreted with caution. The complete correlation matrix is reported in Supplementary Table S1.

Based on clinical relevance and the observed correlations, five perinatal outcomes within the ASD group were selected. Each outcome was investigated in separate binary logistic regressions, adjusting for maternal and paternal ages (Table 4). Because the outcomes were pre-specified, no adjustments for multiple tests were applied. By finding children conceived via ART showed higher odds of twin pregnancy (OR = 5.47, 95% CI [1.57–19.07], *p* = 0.008) and low birth weight (OR = 3.93, 95% CI [1.23–12.49], *p* = 0.021). ART was also inversely associated with vaginal delivery (OR = 0.26, 95% CI [0.09–0.78], *p* = 0.015), but it was not a significant predictor of preterm birth (*p* = 0.247) or uncomplicated pregnancy (*p* = 0.138).

After adjusting for parental age, only maternal age was significantly associated with other selected outcomes. Specifically, higher maternal age was linked to lower odds of vaginal delivery (OR = 0.94, 95% CI [0.88–0.998], *p* = 0.042).

By contrast, ART was not a significant predictor of preterm birth (*p* = 0.247) or neonatal well-being (*p* = 0.621).

### 3.4. Comparison Between ART and Non-ART in ASD

As shown in Table 5, ART-conceived children in the ASD group had significantly higher parental ages at delivery (*p* < 0.001, for both maternal and paternal age), a greater frequency of twin pregnancy (*p* < 0.001), and a lower rate of vaginal delivery (*p* < 0.001) and lower gestational age (*p* < 0.001). Low birth weight was more frequent in the ART group than in the non-ART group (*p* = 0.005).

No significant differences were found between ART and non-ART groups for preterm birth or neonatal well-being (all *p* > 0.05). After Bonferroni correction (α = 0.0056), only the associations with parental age, twin pregnancy, and vaginal delivery remained statistically significant.

Relative risk estimates indicated that ART children had a 34% higher risk of twin pregnancy (RR = 1.34, 95% CI 1.07–1.68) and a 38% lower rate of vaginal delivery (RR = 0.62, 95% CI 0.50–0.76).

## 4. Discussion

### 4.1. Main Findings

In this clinically referred cohort of children with neurodevelopmental disorders (NDDs), conception through assisted reproductive technologies (ART) was more frequent among those diagnosed with autism spectrum disorder (ASD) compared with other NDDs (nASD) (13.2% vs. 5.9%; RR = 1.64). Within the ASD subgroup, ART was associated with twin pregnancy, lower frequency of vaginal delivery, higher rates of low birth weight (LBW), and older parental ages.

Logistic regression analyses confirmed the associations between ART and twin pregnancy, LBW, and cesarean delivery, but not those with preterm birth or neonatal well-being. These findings suggest that ART is not an independent causal exposure but co-occurs with a cluster of perinatal and familial factors influencing neurodevelopmental risk.

### 4.2. Comparison with the Literature

Our results align with previous evidence reporting a modest association between ART and ASD. Liu and colleagues [13] found a 35% increased ASD risk in ART-conceived children (RR = 1.35, 95% CI 1.09–1.68), while the meta-analysis by Andreadou et al. [15] confirmed a significant but attenuated association after adjusting for multiple pregnancies (RR = 1.11, 95% CI 1.03–1.19). Similarly, Rönö and colleagues [11], using Nordic registry data, reported higher rates of ASD and other developmental disorders among ART-conceived children.

Our crude relative risk (RR = 1.64) is higher than these population-based estimates, likely reflecting referral bias in a tertiary clinical sample and the overrepresentation of obstetric complications in ART cases. Importantly, as in previous reviews [9,10], ART in our cohort clustered with multiple pregnancy, LBW, and cesarean delivery.

Fountain and colleagues [16] showed that the ART–ASD association is substantially attenuated after accounting for multiple pregnancies and perinatal complications. Winter et al. further demonstrated that multiple pregnancy, preterm birth, and cesarean collectively explained nearly 78% of the association [17]. Consistently, in our data, ART predicted LBW and cesarean delivery even after adjusting for parental age, underscoring the mediating role of perinatal factors.

### 4.3. Role of Parental Age

ART was also strongly linked to higher maternal and paternal age. This was expected given demographic trends, and is consistent with large-scale cohort studies showing advanced parental age as an independent ASD risk factor. Idring et al. reported that paternal age above 50 years conferred a threefold increase in ASD risk [18], while D’Onofrio et al. extended these findings to other psychiatric and developmental outcomes [19]. Our results support the view that parental age may act both as a confounder and a mediator in the ART–ASD relationship.

Advanced parental age has been associated with increased germline mutation rates and meiotic errors, leading to a higher likelihood of de novo variants and chromosomal abnormalities implicated in ASD. While paternal age mainly contributes through the accumulation of point mutations, advanced maternal age is more often linked to aneuploidy and epigenetic alterations, highlighting distinct yet convergent biological pathways [20,21].

Beyond chronological age, parental genetic and phenotypic characteristics may further contribute to the observed clustering between ART and ASD. Couples who pursue ART might represent a subgroup with specific psychosocial and genetic profiles, including higher polygenic liability for neurodevelopmental conditions or subtle traits of the broader autism phenotype (BAP) [22,23]. In this view, ART could amplify pre-existing familial predispositions rather than act as an independent etiological factor.

### 4.4. Biological Plausibility

The mechanisms suggested by our findings are consistent with a multifactorial model:

- **Multiple pregnancy → preterm birth/LBW → perinatal stressors** such as inflammation, hypoxia-ischemia, and white-matter alterations that increase vulnerability to atypical neurodevelopment [12].
- **Cesarean delivery**, while not likely causal, may reflect underlying obstetric risks common in ART and multiple gestations [9,10].
- **Advanced parental age** as a genetic/epigenetic risk factor, especially paternal age [18,19].

Beyond perinatal factors, several biological pathways have been hypothesized to mediate potential effects of ART procedures on offspring neurodevelopment. Epigenetic alterations, including changes in DNA methylation and genomic imprinting during gamete manipulation, in vitro culture, or cryopreservation, have been reported in ART-conceived children compared with naturally conceived peers [24,25]. Although these findings remain preliminary, such mechanisms may represent indirect procedural contributors rather than primary causes of neurodevelopmental disorders.

### 4.5. Clinical and Public-Health Implications

Our findings support a risk-management rather than an exposure-avoidance approach. Evidence from the European Society of Human Reproduction and Embryology indicates that elective single-embryo transfer (eSET), in combination with optimised laboratory practice and cryopreservation, is effective in reducing multiple gestations while preserving cumulative live-birth rates [26]. Although our cohort is clinically referred, population-based studies demonstrate that selection bias in large pregnancy cohorts tends to influence prevalence estimates more than exposure–outcome associations, supporting the plausibility of our findings [27,28,29].

### 4.6. Strengths and Limitations

Strengths of this study include the relatively large clinically referred cohort (*n* = 507), the use of standardized ICD-10 diagnoses, and the availability of detailed pregnancy, perinatal, and family histories. Rigorous statistical methods were applied with correction for multiple comparisons, increasing the robustness of the findings.

Several limitations should also be acknowledged. First, the ASD group was larger than the nASD group, which may have affected group comparisons. Second, data were collected retrospectively from pre-existing databases, resulting in incomplete parental and individual medical histories in the nASD group and preventing some in-depth analyses. Compared with population-based registry studies, clinically referred cohorts such as ours are likely to over-represent children with developmental concerns or perinatal complications, as these conditions frequently prompt referral to tertiary services. This selection bias may inflate the apparent prevalence of ART-related obstetric complications, thereby limiting external validity. The absence of a population-based control group without neurodevelopmental disorders further limited the possibility of causal inference. In addition, some potentially relevant variables, such as parental smoking and detailed ART characteristics (e.g., IVF vs. ICSI, fresh vs. frozen cycles, infertility etiology), were unavailable. Finally, regression analyses were designed to test ART as a predictor of perinatal factors within ASD, rather than to fully model ART–ASD associations via mediation.

Future studies should aim to collect more comprehensive clinical and perinatal data, ensure balanced representation across groups, and include control populations in order to strengthen causal inference and clarify the mechanisms linking ART, perinatal complications, and neurodevelopmental outcomes.

### 4.7. Future Directions

Future research should use population-based registries with ART linkage for mediation analyses, as in Fountain et al. [16] and Winter et al. [17]. Designs such as sibling-comparison and donor-gamete approaches [19] would further clarify causality. Clinically, evaluating structured developmental surveillance protocols in ART-conceived infants with perinatal risk would be an important next step.

Beyond parental and perinatal variables, ART pregnancies often receive closer obstetric surveillance, potentially increasing exposure to diagnostic procedures such as repeated ultrasound examinations [9]. Although evidence of causal neurodevelopmental effects remains inconclusive, some authors have hypothesized that increased ultrasound exposure might interfere with early neuronal migration [30], representing a potential, albeit unproven, environmental contributor that warrants cautious consideration in future population-based studies.

Additionally, cross-national comparisons between regions with varying ART utilization rates could help clarify whether the observed associations reflect population-level factors, healthcare practices, or true etiological differences in neurodevelopmental outcomes.

Integrating genomic and epigenomic data in future ART cohorts will also be essential to better disentangle inherited predispositions from procedure-related effects, particularly in the context of emerging technologies such as preimplantation genetic testing (PGT) and embryo selection.

## 5. Conclusions

Pregnancies conceived using Assisted Reproductive Technologies (ART) are frequently associated with advanced parental age, multiple gestations, and adverse perinatal outcomes such as preterm birth and low birth weight. In our clinically referred cohort, ART was more common among children with ASD compared with other NDDs, but within ASD, it clustered with multiple pregnancy, LBW, cesarean delivery, and parental age. Together with the existing literature, these findings suggest that the ART–ASD association is largely explained by familial and perinatal mediators rather than ART itself. Clinical emphasis should therefore be placed on reducing multiple gestations, optimizing obstetric care, and ensuring early developmental monitoring for ART-conceived children. As this was a retrospective clinical cohort study without a population-based control group, our findings should not be interpreted in causal terms, but rather as evidence of clustering between ART conception and perinatal risk factors within ASD.

## Figures and Tables

**Table 1:** ART and non-ART conceptions among children with NDDs.

Group	ART	Non-ART	Total
ASD	31	203	234
nASD	16	257	273
Total	47	460	507

ART = Assisted Reproductive Technologies group; non-ART = non-Assisted Reproductive Technologies group; ASD = Autism Spectrum Disorder group; nASD = non-Autism Spectrum Disorder group.

**Table 2:** Distribution of ART conception across diagnostic groups.

Comparison	χ^2^	*p*-Value	RR [95% CI]	OR [95% CI]
ASD vs. nASD	8.17	0.004	1.64 [1.09–2.46]	2.45 [1.31–4.61]

ASD = Autism Spectrum Disorder group; nASD = non-Autism Spectrum Disorder group; RR = relative risk; OR = odd ratio; *p* < 0.05.

**Table 3:** Medical and family history of the ASD group by mode of conception.

Variables	ART (*n* = 31)	Non-ART (*n* = 203)
Sex (M:F)	24:7	161:42
Twin pregnancies, *n*	9	10
Uncomplicated pregnancy, *n*	24	146
Vaginal delivery, *n*	5	98
Preterm birth, *n*	7	26
Neonatal well-being, *n*	25	167
Low birth weight, *n*	8	18
History of miscarriage, *n*	4	24
≥2 miscarriages, *n*	3	4
Maternal medical conditions, *n*	6	41
Maternal medication during pregnancy, *n*	6	41
Family history of NDD, *n*	8	64
Family history of neuropsychiatric disorders—mother, *n*	4	61
Family history of neuropsychiatric disorders—father, *n*	5	56
Family history of neuropsychiatric disorders—siblings, *n*	1	17
Birth weight, grams (mean ± SD)	2.951 ± 718	3226 ± 564
Gestational age, weeks (mean ± SD)	37.6 ± 2.38	38.8± 2.27
Maternal age, years (mean ± SD)	41.2 ± 4.96	32.4 ± 5.68
Paternal age, years (mean ± SD)	42 ± 5.92	35.9 ± 6.12

ART = Assisted Reproductive Technologies group; non-ART = non-Assisted Reproductive Technologies group; NDD = Neurodevelopmental Disorder.

**Table 4:** Logistic regression models testing the effect of ART on perinatal outcomes in the ASD group (adjusted for maternal and paternal age).

Outcome	OR [95% CI]	*p*-Value
Twin pregnancy	5.47 [1.57–19.07]	0.008 *
Vaginal delivery	0.26 [0.09–0.78]	0.015 *
Low birth weight	3.93 [1.23–12.49]	0.021 *
Preterm birth	1.93 [0.64–5.84]	0.247
Neonatal well-being	0.76 [0.25–2.28]	0.621

OR = odds ratio; CI = confidence interval. * *p* < 0.05.

**Table 5:** Comparison of perinatal and familial factors between ART and non-ART children with ASD.

Variable	Test Statistic	*p*-Value
Maternal age at delivery	*t*(232) = −8.12	<0.001 *
Paternal age at delivery	*t*(231) = −5.21	<0.001 *
Twin pregnancy	χ^2^(1, 234) = 20.9	<0.001 *
Vaginal delivery	χ^2^(1, 234) = 11.3	<0.001 *
Low birth weight	χ^2^(1, 234) = 7.81	0.005
Gestational age	U = 1975	<0.001 *
Mean birth weight	U = 2466	0.053
Preterm Birth	χ^2^(1, 234) = 2.12	0.145
Neonatal well-being	χ^2^(1, 234) = 0.05	0.827

Bonferroni-adjusted α = 0.0056. * = significant after Bonferroni correction.

## Data Availability

The original contributions presented in the study are included in the article; further inquiries can be directed to the corresponding authors. Additional data are not publicly available due to privacy concerns.

## Supplementary Materials

The following supporting information can be downloaded at: https://www.mdpi.com/article/10.3390/pediatric17060118/s1, Table S1: Correlation Matrix Between ART and Perinatal Variables in the ASD Group.

## Abbreviations

The following abbreviations are used in this manuscript:

ASD	Autism spectrum disorder
nASD	Group with neurodevelopmental disorder other than ASD
ART	Assisted reproductive technology
non-ART	Children conceived without ART
NDDs	Neurodevelopmental disorders
LD	Language disorder
MDD	Mixed developmental disorder
