# Supplementary material for: Exploring the Association Between Medically Assisted Reproduction and Autism Spectrum Disorder: Clinical Correlations from a Retrospective Cohort
Source: Pediatr Rep. 2025 Nov 4;17(6):118. doi: 10.3390/pediatric17060118 (PMC12641742; doi:10.3390/pediatric17060118)
Supplement: Supplementary file 1 [file pediatrrep-17-00118-s001.zip › pediatrrep-3908949-supplementary.pdf]

Table S1 Correlation Matrix Between ART and Perinatal Variables in the ASD Group

		Sex	ART	Twin Pregnancy	Uncomplicated Pregnancy	Vaginal Delivery	Preterm birth	Neonatal Well-being	Low birth weight	History of miscarriage	Two or more miscarriages	Maternal medical conditions	Maternal medication during pregnancy	Maternal age at delivery	Paternal age at birth	Family history of neuropsychiatric disorders	Family history of neuropsychiatric disorders - Mother side	Family history of neuropsychiatric disorders - Father side	Family history of neuropsychiatric disorders - Siblings
**Sex**	*r*	—																	
	p	—																	
**ART**	*r*	-0.016	—																
	p	0.811	—																
**Twin Pregnancy**	*r*	-0.039	**0.299***	—															
	p	0.550	< .001	—															
**Uncomplicated Pregnancy**	*r*	-0.080	0.042	**-0.133***	—														
	p	0.222	0.524	0.041	—														
**Vaginal Delivery**	*r*	0.076	**-0.220***	**-0.264***	0.100	—													
	p	0.250	< .001	< .001	0.128	—													
**Preterm birth**	*r*	-0.033	0.095	**0.509***	**-0.220***	**-0.211***	—												
	p	0.617	0.147	< .001	< .001	0.001	—												
**Neonatal Well-being**	*r*	0.006	-0.014	**-0.228***	**0.213***	0.033	**-0.322***	—											
	p	0.932	0.828	< .001	0.001	0.612	< .001	—											

**Low birth weight**	*r*	-0.119	**0.18***	**0.492***	**-0.180***	**-0.204***	**0.677***	**-0.437***	—										
	p	0.070	0.005	< .001	0.006	0.002	< .001	< .001	—										
**History of miscarriage**	*r*	-0.004	0.011	-0.061	-0.099	**-0.141***	-0.074	0.001	-0.088	—									
	p	0.946	0.864	0.350	0.132	0.031	0.261	0.989	0.178	—									
**Two or more miscarriages**	*r*	0.090	**0.153***	0.040	-0.117	-0.105	0.001	-0.049	-0.062	**0.476***	—								
	p	0.168	0.019	0.546	0.073	0.109	0.989	0.459	0.344	< .001	—								
**Maternal medical conditions**	*r*	-0.074	0.119	-0.012	**-0.212***	-0.097	0.046	-0.053	0.020	0.032	0.057	—							
	p	0.258	0.069	0.849	0.001	0.141	0.482	0.417	0.759	0.623	0.384	—							
**Maternal medication during pregnancy**	*r*	0.022	-0.007	0.007	**-0.219***	-0.101	0.073	-0.016	-0.008	**0.144***	**0.162***	**0.634***	—						
	p	0.737	0.914	0.913	< .001	0.124	0.268	0.811	0.909	0.028	0.013	< .001	—						
**Maternal age at delivery**	*r*	-0.088	**0.471***	**0.190***	-0.097	**-0.178***	0.055	0.032	0.070	0.109	0.095	**0.169***	0.120	—					
	p	0.181	< .001	0.004	0.140	0.006	0.402	0.626	0.287	0.096	0.149	0.009	0.067	—					
**Paternal age at birth**	*r*	-0.065	**0.325***	0.112	-0.033	-0.049	0.068	-0.032	0.090	**0.139***	0.016	0.116	0.078	**0.680***	—				
	p	0.324	< .001	0.087	0.612	0.458	0.299	0.628	0.172	0.034	0.802	0.078	0.234	< .001	—				
**Family history of neuropsychiatric disorders**	*r*	-0.017	**-0.128***	-0.051	-0.077	-0.029	0.026	0.112	-0.103	-0.018	-0.059	0.078	0.058	-0.007	-0.032	—			
	p	0.796	0.051	0.442	0.239	0.663	0.693	0.086	0.115	0.783	0.369	0.233	0.377	0.911	0.629	—			
**Family history of neuropsychiatric disorders - Mother side**	*r*	-0.009	**-0.130***	-0.045	-0.069	0.007	0.023	0.066	-0.037	0.007	0.003	0.014	0.046	-0.010	-0.031	**0.681***	—		
	p	0.890	0.047	0.497	0.293	0.909	0.728	0.313	0.572	0.921	0.962	0.830	0.481	0.874	0.638	< .001	—		
**Family history of neuropsychiatric disorders - Father side**	*r*	-0.101	-0.088	-0.070	0.037	0.062	-0.017	0.100	**-0.148***	0.021	-0.047	0.039	0.042	-0.007	-0.042	**0.653***	**0.284***	—	

	p	0.123	0.177	0.289	0.576	0.347	0.798	0.127	0.024	0.749	0.473	0.555	0.518	0.916	0.523	< .001	< .001	—	
**Family history of neuropsychiatric disorders - Siblings**	*r*	0.109	-0.066	0.032	-0.111	-0.062	0.021	0.010	0.051	-0.057	-0.051	-0.003	0.015	-0.050	-0.046	**0.317***	0.036	-0.062	—
	p	0.096	0.318	0.630	0.091	0.344	0.746	0.883	0.437	0.385	0.440	0.967	0.815	0.444	0.487	< .001	0.586	0.346	—

Sex = Sex of the child (1 = male, 0 = female); Assisted reproductive technology (ART) = Conception through ART procedures (present/absent); Twin pregnancy = Pregnancy resulting in twins (present/absent); Uncomplicated pregnancy = Pregnancy without complications (present/absent); Vaginal delivery = Vaginal delivery (present/absent; absent = cesarean section); Preterm birth = Birth before 37 weeks of gestation (present/absent); Neonatal well-being = Satisfactory neonatal health status at birth (present/absent); Low birth weight = Birth weight < 2500 g (present/absent); History of miscarriage = One or more miscarriages before the index pregnancy (present/absent); Two or more miscarriages = History of ≥ 2 miscarriages (present/absent); Maternal medical conditions = Presence of pre-existing maternal medical conditions (present/absent); Maternal medication during pregnancy = Use of pharmacological treatment during the pre- or perinatal period (present/absent); Maternal age at delivery = Maternal age in years at the time of delivery; Paternal age at birth = Paternal age in years at the time of birth; Family history of neuropsychiatric disorders = Presence of neuropsychiatric disorders in first-degree relatives (present/absent); Family history of neuropsychiatric disorders – mother = Presence of neuropsychiatric disorders in the maternal lineage (present/absent); Family history of neuropsychiatric disorders – father = Presence of neuropsychiatric disorders in the paternal lineage (present/absent); Family history of neuropsychiatric disorders – siblings = Presence of neuropsychiatric disorders in siblings (present/absent). * $p < 0.05$.
